# Cognitive Gains of Aerobic Exercise in Patients With Ischemic Cerebrovascular Disorder: A Systematic Review and Meta-Analysis

**DOI:** 10.3389/fcell.2020.582380

**Published:** 2020-12-18

**Authors:** Yimei Shu, Qing He, Yi Xie, Wanrong Zhang, Shuang Zhai, Ting Wu

**Affiliations:** ^1^Department of Neurology, The First Affiliated Hospital of Nanjing Medical University, Nanjing, China; ^2^Department of Neurology, Xuzhou First People's Hospital, The Affiliated Xuzhou Municipal Hospital of Xuzhou Medical University, Xuzhou, China

**Keywords:** stroke, cognition, aerobic exercise, systematic review, meta-analysis, ischemic cerebrovascular disease

## Abstract

**Background:** Cognitive impairment has become an important problem in ischemic cerebrovascular disorder survivors as disease related deaths have been significantly reduced. Aerobic exercise, the most prevalent mode of physical activity, positively contributes to cognition in both healthy population and people with cognitive impairment. However, studies on its associations with cognitive gains in patients with ischemic cerebrovascular disease showed mixed findings.

**Objective:** To explore the cognitive effects of aerobic exercise on ischemic cerebrovascular disorder survivors and investigate the possible moderators on exercise benefits.

**Method:** Randomized controlled trials investigating the effects of sole aerobic exercise on cognitive function in population with ischemic intracranial vascular disorder compared to any control group who did not receive the intervention were enrolled in this systematic review and meta-analysis. Four online database (Pubmed, Cochrane Library, Embase, and Web of Science) were searched.

**Results:** The initial search returned 1,522 citations and ultimately 11 studies were included in the systematic review. Analysis of seven studies showed the beneficial but not statistically significant impact of aerobic exercise on global cognitive function (0.13; 95% Cl −0.09 to 0.35; *p* = 0.25). Participants already with cognitive impairment benefited more from this intervention (0.31; 95% Cl 0.07–0.55; *p* = 0.01) and moderate intensity might be the optimal choice (0.34; 95% Cl −0.01 to 0.69; *p* = 0.06). The program duration and initiation time after stroke occurrence did not predict better cognitive outcome. Aerobic exercise was not associated with improvement of processing speed and executive function, the two subdomains of cognitive function.

**Conclusions:** Aerobic exercise may contribute to cognitive gains in survivors of ischemic cerebrovascular disorder, especially for population already with cognitive decline. Our findings suggest that the adoption of moderate intensity aerobic exercise might improve cognition in such population.

## Introduction

Cerebrovascular disorder is one of the most prevalent diseases in old adults. With the reduction of fatal risk, the incidence of cognitive impairment or dementia in survivors of such kind of disease has also increased, exerting heavy burden on family and the society (Hurd et al., 2013). Vascular dementia, the most severe stage of cognitive impairment derived from vascular factors, is the most common cause of dementia second only to Alzheimer's disease (Gorelick et al., 2011). Stroke is the most important and prevalent disease type of ischemic cerebrovascular disorder and approximately 30% of the survivors are estimated to develop dementia. The risk is three to five times greater than those without any stroke history (Kalaria et al., 2016). Recent evidence also suggests the central role of small blood vessel disease in vascular dementia development (Dichgans and Leys, 2017; Wallin et al., 2018). The development of cognitive impairment from the occurrence of ischemic cerebral vascular disease is complex as vascular, neurodegenerative or mixed processes are involved (Kalaria et al., 2016). The variations such as disease type, location, number of lesions, and severity all make the symptoms complicated. The location, in particular, is closely associated with specific cognitive domains. For instance, the prefrontal and parietal circuits is associated with executive function (Colcombe and Kramer, 2003) while the frontal lobe is in charge of working memory and processing speed (Stephens et al., 2004). At present, the main strategy to manage cognitive decline after ischemic cerebrovascular disorder is prevention. However, evidence is still controversial among the preventive strategies (Van der Flier et al., 2018).

Apart from improving physical fitness, increasing evidence suggests the strong link between aerobic exercise and brain cognitive health. Aerobic exercise is beneficial to alleviate hypertension, atherosclerosis, diabetes mellitus, high cholesterol, which are all potential contributors to cerebrovascular disease and vascular cognitive deterioration (Sahathevan et al., 2012). One of the molecular mechanisms is the protection effect on endothelium against oxidative stress and inflammation (Palmefors et al., 2014; Luca and Luca, 2019). As the consumption of oxygen and energy is high in brain, chronic cerebral hypoperfusion is a crucial promotor of cognitive dysfunction (Duncombe et al., 2017) and aerobic exercise can attenuate this reduction (Moraine et al., 1993). However, whether the vascular benefits of aerobic training can predict cognitive gains still needs further exploration. Neurogenesis is one of the processes associated with improved cognition (Van Praag et al., 1999). Aerobic training is also positively associated with synaptic plasticity, the essential biological change for memory, and learning. It is involved in both long-term potentiation enhancement and long-term depression induction (Bettio et al., 2019). Neuron density and gray matter volumes are increased in hippocampus, prefrontal and cingulate cortices after physical activity (Ruscheweyh et al., 2011; Kleemeyer et al., 2016). Moreover, Aerobic exercise is found to positively impact several important neurotransmitters and neurotrophic factors, including dopamine (Loprinzi et al., 2013), brain-derived neurotrophic factor (BDNF) (Vaynman et al., 2004), vascular endothelial growth factor (VEGF) (Ding et al., 2006b) and insulin-like growth factor-1 (IGF-1) (Ding et al., 2006a).

Several exercise modes have been practiced in hope of improving cognitive functions in ischemic cerebrovascular disorder survivors (Schmidt et al., 2013; Tiozzo et al., 2015; Han et al., 2017). Here, we conduct a meta-analysis review on the cognitive effects of aerobic exercise in patients with ischemic cerebrovascular disorder. We also explore the modulation of population and exercise characteristics on the training efficacy. This analysis can be provided important implication for ways to maximize cognitive benefits.

## Methods

We conducted the systematic review and meta-analysis complying with established guidelines from Preferred Reporting Items for Systematic Reviews and Meta-Analysis (PRISMA).

## Search Strategy

We searched the electronic database Pubmed, Cochrane Library, Embase and Web of Science for clinical trials published in English up to October 2020. The search strategy was composed of the following items: (cerebrovascular disorder) OR (cerebral OR brain OR cranial OR intracranial) AND (vascular OR vessel OR hemorrhage OR infarction OR stroke) AND (cognition OR cogniti^*^ OR neuropsychological OR attention OR memory OR processing OR language OR visuospatial OR executive function OR dementia) AND (aerobic OR endurance OR exercise). Terms were searched in “All Fields” and medical subject headings (MeSH) was also used for “cerebrovascular disorder,” “cognition” and “stroke.” Reference lists had also been examined to look up for other possibly relevant articles.

## Study Selection

Studies were included if they met all of the following criteria: (1) Participants with ischemic cerebrovascular disorder which were confirmed by clinical evidence. Studies focusing on participants with rick factors for intracranial ischemia like diabetes or high blood pressure but without structured vascular impairment in brain were excluded. There was no limitation on the baseline cognitive status. However, studies including samples with other neurological or mental disease were ruled out. (2) Monitored and structured aerobic exercise, regardless of intensity, duration or frequency. The training program should last for at least 4 weeks. Studies combining aerobic exercise with other physical intervention (for example, stretching exercise) were also included if aerobic exercise was the only difference in intervention between groups. (3) A supervised control group with usual care, educational program or other physical training mode except aerobic exercise. Educational program should not specifically provide information about physical activity. (4) At least one valid neuropsychological test of cognitive function with data available before and in the end of intervention. (5) Randomized controlled trials.

## Data Collection and Extraction

Dr. Shu performed the initial search, removed the duplicates and cleared out the apparent non-related articles according to title and abstract. The abstract of the remaining studies were assessed independently by Dr. He and Dr. Xie and full text was obtained for possibly eligible studies. Then, Dr Shu and Dr He reviewed the full text of each article independently in accordance with the inclusion and exclusion criteria. Conflicts were discussed between the researchers and consensus was reached. Information on participants, strategy for intervention and control groups, cognitive outcome were extracted by one researcher using a standardized form. We extracted mean, standard deviation and number of participants of each assessment from all eligible studies. We used final measurement values for analysis as most studies did not provide the change from baseline. Missing final measurement value was calculated if baseline measurement values and the changes were provided, using the formula: SD_final_= $\frac{2\times \text{R}\times \text{S}{\text{D}}_{\text{base}}+\sqrt{4\times {\text{R}}^{2}\times {\text{SD}}_{\text{base}}^{2}-4\times {\text{SD}}_{\text{base}}^{2}+4\times {\text{SD}}_{\text{change}}^{2}}}{2}$, *R* = 0.5. The value for *R* was based on the assumption that there was a moderate correlation between measures before and after intervention. This method was proved feasible in previous publications (Gates et al., 2013). When mean and SD were unavailable with sample size (N), median (Q2), first quartile (Q1) and third quartile (Q3) provided, we calculated mean and SD using the formula respectively: mean = (Q1+Q2+Q3) 3, SD = $\frac{\text{Q}3-\text{Q}1}{2\times \text{norminv}\;\left(\frac{0.75\times \text{Q}3-0.125}{\text{Q}3+0.25},0,1\right)}\;$ (Wan et al., 2014; Luo et al., 2018). And in three studies (Bo et al., 2019; Ploughman et al., 2019; Rosenfeldt et al., 2019), subgroup data was provided separately and we obtained the total mean and SD using the formula: mean =$\frac{{\text{N}}_{1}\times {\text{M}}_{1}+{\text{N}}_{2}\times {\text{M}}_{2}}{{\text{N}}_{1}+{\text{N}}_{2}}$, SD = $\sqrt{\frac{\left({\text{N}}_{1}-1\right)\times {\text{SD}}_{1}^{2}+\left({\text{N}}_{2}-1\right)\times {\text{SD}}_{2}^{2}+\frac{{\text{N}}_{1}\times {\text{N}}_{2}}{{\text{N}}_{1}+{\text{N}}_{2}}\times \left({\text{M}}_{1}^{2}+{\text{M}}_{2}^{2}-2\times {\text{M}}_{1}\times {\text{M}}_{2}\right)}{{\text{N}}_{1}+{\text{N}}_{2}-1}}$, M standing for mean value.

## Quality Assessment

The methodological quality was assessed by Dr Xie and Dr Zhang separately in accordance with Cochrane risk-of-bias tool. Seven sections, namely, random sequence generation, allocation concealment, blinding of participants and personnel, blinding of outcome assessment, incomplete outcome data, selective reporting, and other bias were evaluated in each included study and were rated low, unclear (missing or deficient information) or high risk of bias depending on data provided. Disagreement was discussed and consensus was reached.

## Data Synthesis and Analysis

The meta-analysis was carried out using RevMan 5.3 (Review Manager (RevMan) [Computer program]. Version 5.3. Copenhagen: The Nordic Cochrane Center, The Cochrane Collaboration, 2014). We calculated standardized mean difference (SMD) together with 95% Cl, as variant scales were utilized to measure cognitive status. For outcome with larger index indicating worse cognitive condition, it was entered into the software as a negative number. We used Chi^2^ test to assess heterogeneity between trial results. Considering its low power under the circumstance of small sample size or small number of studies included, a *p* value < 0.1 was regarded statistically important between-trial heterogeneity. We also used a random-effects model as the fixed model would offer narrower Cls although the same quantitative conclusions.

We first assessed whether aerobic exercise exerted significant and positive effect on global cognition. Subgroup analysis was conducted to explore whether characteristics of participants, intervention and measurement moderated the influence. For population characteristic, we mainly focused on baseline cognitive status (cognitive impairment or normal cognitive status). Cognitive impairment was defined as Addenbrooke's Cognitive Examination-Revised (ACER) Score < 82 or Montreal Cognitive Assessment (MoCA) Score < 26. Characteristics of intervention included intensity (light or moderate or vigorous, Billinger et al., 2014), duration (< 3 months or ≥ 3 months), procedures for control group (physiotherapy or standard care without extra exercise intervention). The trials employing high intensity training [defined as exercise with maximal or near maximal heart rate or oxygen uptake Bonsu and Terblanche, 2016; Riebe, 2018] were excluded as recent evidence uncovered different impacts this special mode could exert on cardiovascular disease risks from aerobic training (Hasegawa et al., 2018). We then investigated the moderating effect of measurement used (subjective or objective). We also limited the disease type to stroke to uncover effects of aerobic exercise on global cognitive function and did the subgroup analysis based on the starting point of intervention poststroke (< 3 months or ≥ 3 months). In the meantime, change of specific domains of cognition before and after aerobic exercise was explored, including processing speed and executive function. The latter was further divided into response inhibition, set shifting and working memory. A *p* value < 0.05 was considered statistically significance. Inconsistency between subgroups was shown in terms of *I*^2^ statistic in the forest plot.

## Results

### Characteristics of Included Studies

**Figure 1:**
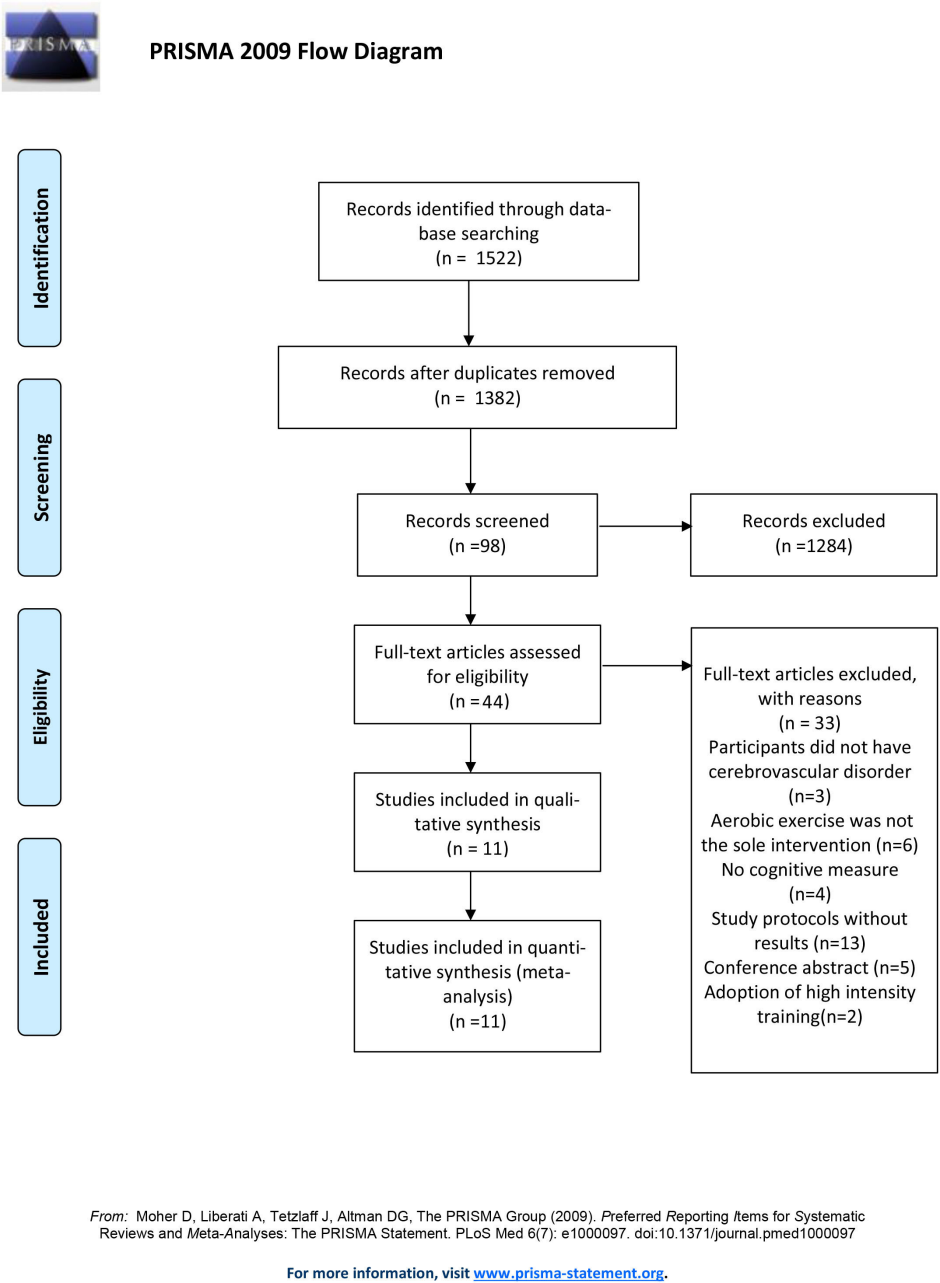
PRISMA flow chart of study selection process.

The selection process is summarized in the PRISMA study flow diagram (Figure 1). The initial search returned 1,522 potentially relevant citations and was reduced to 1,382 following duplicates removal. A total of 80 articles were retrieved for full-text review. There were two studies producing more than one publication (Tang et al., 2014, 2016; Liu-Ambrose et al., 2016; Hsu et al., 2017, 2018; Dao et al., 2019; Khattab et al., 2019), they were regarded as one study in the quantitative analysis. One publication (Rosenfeldt et al., 2019) was the secondary analysis of two randomized controlled trials (ClinicalTrials.gov registration numbers NCT02076776, National Institutes of Health, and NCT02494518, American Heart Association). As it was the only publication relevant to the two trials and provided more data interested than that on the official webpage of ClinicalTrials.gov, we considered the two trials as one study. Finally, 11 studies were included in the quantitative analysis, representing data from 1,038 participants. Studies were published between November 2009 and February 2020. Study sizes ranged from 30 to 362 and the average age was 71.58. There was no significant difference in age between intervention (66.1; SD:12) and control groups (68.2; SD:10.8). Patients were diagnosed with stroke in 10 studies (Quaney et al., 2009; El-Tamawy et al., 2014; Liu-Ambrose et al., 2016; Tang et al., 2016; Ploughman et al., 2018; Bo et al., 2019; Debreceni-Nagy et al., 2019; Ihle-Hansen et al., 2019; Nave et al., 2019; Rosenfeldt et al., 2019) and with small vessel ischemic disease in the rest one (Liu-Ambrose et al., 2016). Among the aerobic exercise interventions, one study maintained participants' heart rate reserve (HRR) below 40% (Debreceni-Nagy et al., 2019) and three studies adopted moderate intensity training (El-Tamawy et al., 2014; Liu-Ambrose et al., 2016; Nave et al., 2019). The remaining studies adopted vigorous intensity intervention. Except for the trial done by Ihle-Hansen et al., the training frequency was three or five times per week. Ihle-Hansen and his colleagues adopted the strategy to perform the vigorous exercise once a week with a 30-min physical activity every day. The entire program lasted for 1–18 months. The baseline information and methodological characteristics of these studies are summarized in Tables 1, 2.

**Table 1:** Characteristics of included studies.

**Study**	**Population characteristics**	**Number of participants (male/female) and drop-out**	**Intervention**	**Control**	**Cognitive outcome(s)**
^*^Debreceni-Nagy et al. (2019)	With the age of 18~75; ischemic or haemorrhagic stroke more than 3 months ago; no dementia (MMSE score > 23)	Intervention = 19(13/6), one withdrew consent and one died because of pulmonary embolism during a resting, weekend day Control = 16(11/5), no drop-out	A conventional, customized physiotherapy for 30 min followed by a bicycle training for 30 min including a warm-up (5 min), a therapy (20 min), maintenance of target heart rate and a cool-down (5 min) phase. HRR was maintained between 30 and 40%. The program was performed on 20 cosecutive weekdays	The same physiotherapy for 60 min	Global cognitive function (FIM cognitive subtest); processing speed (Symbol Search and coding; Wechsler, 2008); working memory (WAISIV, Digit Span)
El-Tamawy et al. (2014)	Egyptian stroke patients with strokes in the territory of anterior circulation; time interval between stroke occurrence and intervention initiation ranging from 3 to 18 months; cognitive impairment (ACER score < 82)	Intervention = 15 (11/4), no drop-out; Control = 15(10/5), no drop-out	A designed physiotherapy program consisting of stretching exercises, facilitation for weak muscles, strengthening exercise, postural control and balance, functional training and gait training. This program lasted for 25–30 min per session. It was followed by a rest for 10~15 min and then a bicycle training for 40~45 min including a warm-up (5~10 min), an active phase (30 min) and a cool-down (5 min). The program was considered moderate intensity and was performed three times per week with the total duration for 8 weeks. It was instructed by a qualified physiotherapist	The same physiotherapy program for 25~30 min with equivalent frequency and total duration	Global cognitive function (ACER) with subsets available including attention/processing speed, delayed memory, verbal fluency, language and visuospatial ability
^*^Ihle-Hansen et al. (2019)	Aged over 18; first- ever or recurrent stroke (infarction or intracranial haemorrhage), 10–16 weeks post the occurrence; community-dwelling with modified Rankin Scale score < 5, MMSE ≥ 21 (or ≥ 17 for patients with aphasia)	Intervention = 177 (99/78), nine died; Control = 185(120/65), nine died. The death was no correlation with the intervention	A 45–60 min physical exercise including 2–3 bouts of vigorous activity (15–17 on the Borg scale of perceived exertion) every week and 30-min-physical activity 7 days a week. The intervention did not include supervised or observed training but the amount and intensity of each day's activities were recorded. The program lasted for 18 months	Standard care	Global cognitive function (MMSE), processing speed (TMT-A), set-shifting (TMT-B)
^#^Liu-Ambrose et al. (2016)	Aged over 18; with diagnosis of small vessel ischemic disease; cognitive impairment (MoCA score < 26 and MMSE score ≥ 20)	Intervention = 35(16/19), two withdrew and the other one failed to finish the cognitive assessment; Control = 35(18/17), 7 withdrew and one missed assessment	A 60-min class including a 10-min warm-up, a 40-min walk, and a 10-min cool down. The training intensity was monitored based on the measure of HRR (progressing from 40 to 60~70% in first 12 weeks and maintaining at 65%), RPE (14~15), “talk” test (a pace at which conservation was difficult). The intervention was instructed and class attendance was recorded	Usual care as well as monthly educational materials about vascular cognitive impairment and healthy diet. No specific information about physical activity was provided	Global cognitive function (ADAS-Cog), performance of daily living (ADCS-ADL), global executive function (EXIT-25), response inhibition (Stroop Test), working memory (verbal digit span tests, digits forward minus digits backwards), set shifting (TMT, part B minus part A)
^*^Nave et al. (2019)	Aged over 18; ischemic or hemorrhagic stroke survivors; 5~45 days after stroke occurrence; Barthel index score ≤ 65	Intervention = 105(60/45), two were excluded before training, one violated the strategy, five had severe adverse event, four had adverse event, four were transferred to other clinic, four refused to participate; Control = 95(59/36), one violated the strategy, one stopped training more than 5 days after initiation, three had severe adverse event, one had adverse event, one refused to participate, four were transferred to other clinic	A therapist led session for 50 min, including 25 min of core intervention (training aimed at target heart rate). The training was treadmill based with bodyweight support. The intensity was maintained at 50~60% of maximum heart rate. The frequency was five times per week and the total duration was 4 weeks. The training was monitored	A therapist led session for 50 min, including 25 min of core intervention. The core part focused on contraction and relaxation of muscle groups in the face, arms, shoulders, back, and abdomen. The intervention was conducted with the same frequency and duration and was also monitored	Global cognitive function (MoCA); processing speed (TMT A); set shifting (TMT B)
^*^Ploughman et al. (2019)	Aged over 18; ischemic or hemorrhagic stroke >6 months; able to perform two-step instruction, ambulation with/without aid ≥10 m, without high-risks; self-reported cognitive problems related to stroke interfering with daily functioning but without moderate/severe receptive aphasia	Intervention = 25 (16/9), Control = 27(20/7). three unable to secure travel arrangements, four unable to comply with time requirement, one refusing group assignment	Aerobic exercise performed on a treadmill with body weight support and target heart rate zone corresponding to 60–80% of peak oxygen uptake (VO_2peak_). The program was performed three times/week for 10 weeks and was instructed.	Therapeutic activity including interventions designed to improve range of motion and comfort of the affected side (massage and active and passive range of motion exercises) and to relearn routine mobility tasks such as lying to sitting, rolling, sit to stand, and standing balance (functional task training)	Global cognitive function (Fluid Intelligence)
^#^Quaney et al. (2009)	Chronic stroke survivors (≥ 6 months prior to the intervention); with residual hemiparetic deficits in either the upper or lower extremity; MMSE > 23	Intervention = 19(10/9); Control =19(7/12) Two patients dropped out after enrollment and were not included in the analysis	A 55-min session including a 5-min warm-up, a 45-min aerobic exercise using a stationary bicycle and a 5-min cool-down. Intensity gradually progressed from 40% maximum heart rate in the first 10~20 min in week 1 to 70% maximum heart rate. It was performed three times per week for 8 weeks under the physician's supervision	A 45-min upper and lower extremity stretching activities, 3 times per week or 8 weeks (24 sessions) at home. Physical therapist asked the participants about exercise questions every week	Response inhibition (Stroop Task); working memory (WCST); set shifting (TMT, part B minus part A)
^*^Rosenfeldt et al. (2019)	Aged 18~85; single, unilateral chronic stroke survivors (≥6 months prior to intervention); Fugl-Meyer Motor Score 19~55 in involved upper extremity	Intervention = 32(22/10), one dropped out with poor skin integrity, three with non-compliance, one with recurrent stroke; Control = 8(7/1), one dropped with non-compliance and two with unrelated injury	A 45-min cycling consisting 5-min warm-up,35-min main exercise set, and a 5-min cool-down. HRR was maintained 60~80%. The aerobic exercise was also combined with a 45-min upper extremity repetitive arm exercises. It was conducted three times per week for 8 weeks. Heart rate was continuously monitored.	A 45-min session of stroke-related education followed by an identical 45-min session of upper extremity repetitive arm exercises, with equivalent frequency and duration	Global cognitive function (SIS 3.0) with subsets available including memory, language and activities of daily living
^#^Tang et al. (2014)	Aged 50–80; >1 year post-stroke, completing stroke rehabilitation, living in the community, and able to walk 5 m independently with or without assistive devices	Intervention = 25(14/11), three dropped out; Control = 25(15/10), no drop-out	Exercise including 10-min warm up, 0~40 min aerobic component and 10-min cool down. Training modes included brisk level and inclined overground walking, upright and recumbent cycle ergometry, and non-traditional forms of exercise utilizing functional movements, such as marching-on-the-spot, repeated sit-to-stand, and step-ups onto platform steppers. The intensity progressed to 58.1% HRR and RPE 14.2 (a little bit hard to moderate) by the end of the intervention. The program was conducted three times per week for 6 months. The intervention was instructed	Stretching, weight bearing, postural awareness and balance exercises with HRR < 40% and with equivalent frequency and duration	Response inhibition (Color-Word Stroop Test); working memory (The Verbal Digit Span Backwards Test); set shifting (TMT B)
^*^Bo et al. (2019)	Aged over 18; < 6 months post-stroke; without severe somatic diseases or mental disorders, without visual or auditory disturbances in recent months; meeting the diagnostic criteria for vascular cognitive impairment (criteria from National Institute of Neurological Disorders and Stroke-Canadian Stroke Network)	Intervention = 86(50/39), 25 dropped out, 13 declined, 12 felt unwell; Control = 91(52/39), 21 dropped out; 9 declined, 13 felt unwell; the exclusion had no correlation with the intervention	Exercise including a 5–10 min warm-up period of aerobic exercise, such as jogging or cycling, followed by the main component (30–35 min) of endurance, strength, and balance exercise, ending with a 5-min cool-down of stretching and exercises to recover normal cardiac levels. The intensity was maintained at the moderate intensity level with the Borg scale of 13–15. The program was performed three times per week, lasting for 12-weeks. Participants' heart rates were monitored throughout the session	Usual care and a 45-min video documentaries three times per week, for 12 weeks	Spatial imagination (The mental rotation test), response inhibition (Stroop test), working memory (Digit Span Test- Forward), set shifting (TMT-B)

*ACER, Addenbrooke's Cognitive Examination-Revised; ADAS-Cog, Alzheimer's Disease Assessment Scale-Cognitive section; ADCS-ADL, Alzheimer's Disease Cooperative Study–Activities of Daily Living; EXIT, executive interview; HIT, high intensity training; HIIT, high intensity interval training; HRR, heart rate reserve; MoCA, Montreal Cognitive Assessment; MMSE, Mini -mental State Examination; RPE, Borg Rating of Perceived Exertion; SIS, Stroke Impact Scale; SSS, Scandinavian Stroke Scale; TMT, Trail- Making Tests; WAIS-IV, Wechsler Adult Intelligence Scale-Fourth Edition; WCST, Wisconsin Card Sorting Task*.

* *The number of participants refers to the number of participants accomplishing post-intervention assessment*.

# *The number of participants refers to the number of participants receiving the intervention*.

**Table 2:** Comparisons of participants‘ characteristics at baseline.

	**Age Mean (SD)**	**Characteristic of the cerebral infarction involved**	**Basic information**	**Baseline physical strength and movement function**	**Baseline Cognitive Function**	**Other neurological and/or psychological condition/deflects**	**Comorbid Conditions**	**Other**
Debreceni-Nagy et al. (2019)	58.7 (11.2)	Months post occurrence, mean (SD):11.4 (13.7); stroke type, ischemic/hemorrhagic:11/7; Times of involvement, first/recurrent stroke, *n*: 29/6; Hemisphere involved, dominant side, *n*: 17; Stroke severity, NIHSS, mean (SD): 3.4 (2.1)	BMI, mean (SD): 27.8 (4.6);	Walking independently, *n*:20; Aerobic fitness (VO2max) (mL/kg/min) mean (SD):12.6 (4.8);	MMSE, mean(SD):27.9(1.4)	Motor aphasia,n:3;	Cardiovascular disorder, n: 14	Sedentary lifestyle (physical activity ≤ 3 ×30 min/week), n:32;
El-Tamawy et al. (2014)	49.0 (6.6)	Months post occurrence: range from 3 to 18 months	BMI, mean (SD): 25.5 (2.0)	—	ACER Score, mean(SD): 74.0 (5.8)	—	—	—
Ihle-Hansen et al. (2019)	72 (11.6)	Days post stroke, mean (SD): 111.7 (21.1); Stroke type, ischemic/hemorrhagic, *n*: 346/34; Stroke severity, NIHSS, mean (SD): 1.6 (2.4); Previous stroke, *n*: 29;	—	Motor Assessment Scale, mean (SD): 41.8 (7.1); Barthel index, mean (SD): 96.2 (0.8); Berg Balance Scale, mean (SD): 2.5 (1.4); Timed Up and Go test(s), mean (SD): 14.2 (23.1); 6-min walk test(m), mean (SD): 390.1 (204.3)	MMSE, mean(SD): 27.8(2.5)	—	Transient ischemic attack, n:38; Myocardial infarction, n:47; Heart failure, n:9;Atrial fibrillation, n:75; Hypertension, n:199; Diabetes mellitus, n:54; Lung diseases, n:44	—
Liu-Ambrose et al. (2016)	74.3 (8.3)	Sub-cortical ischemic cognitive impairment	Weight(kg), mean (SD): 71.2 (14.2); BMI, mean (SD): 25.9 (3.8); Resting heart rate, mean (SD): 68.6 (13.8); Resting systolic blood pressure (mmHg), mean (SD): 136.0 (18.5); Resting diastolic blood pressure (mmHg), mean (SD): 78.5 (10.8)	Functional comorbidity index, mean (SD): 2.8 (1.9); 6-Min Walk Test (m), mean (SD): 494.9 (97.8); Physical activity scale for the elderly, mean (SD): 121.5 (64.7)	MMSE, mean(SD):26.4(2.9); MoCA, mean(SD):21.2(3.9),ADAS, cognition, mean(SD):11.0(5.5)		Hypertensive, n:37;	Education (>high school), n:51; waist to hip ratio, mean(SD):0.9(0.08)
Nave et al. (2019)	69 (12)	Days post stroke, mean (SD): 28.5 (17.2); Stroke type, ischemic/hemorrhagic, *n*: 181/9; Anterior circulation stroke, *n*: 156; Stroke severity, NIHSS, mean (SD): 8.2 (4.9); Previous stroke, *n*:27; Cause of ischemic stroke, *n*: large artery atherosclerosis (17), cardioembolism (18), small vessel occlusion (16), other causes (3), undetermined causes (34), competing causes (3)	—	Maximal walking speed (m/s), mean (SD): 0.5 (0.4); Barthel index score, mean (SD): 48.0 (16.5); 6-Min Walking distance(m), mean (SD): 122.2 (112.3)	MoCA, mean(SD): 23.2(5.7)	Depression (measured by CES-D), mean(SD):10(6.1);	—	—
Ploughman et al. (2018)	63.4 (11.3)	Months post occurrence, mean (SD): 41.0 (39.8); Stroke type, ischemic/hemorrhagic, *n*:40/12; Hemisphere involved, left/right/bilateral, 24/25/3; Stroke severity: NIHSS, mean (SD): 4.9 (4.2)	—	Aerobic fitness (VO_2max_) (mL/kg/min) mean (SD): 16.7 (4.8); Chedoke stage of leg impairment mean (SD): 5.2 (1.5); Walking speed (cm/s) mean (SD): 84.5 (40.9);	MoCA mean(SD):23.8(5.6)	Depression Score (measure by HADS-D) mean(SD):4.6(3.3); Expressive aphasia (number with mild severe according to NIHSS) :15	Hypertension:26; Diabetes:13; Dyslipidemia:9; Cardiovascular disease, including congestive heart failure, abdominal aortic aneurysm, carotid stenosis, and coronary artery bypass graft:6; Other cardiac (eg, aortic valve repair or atrial fibrillation): 5; Kidney disease:3; Myocardial infarction:1	—
Quaney et al. (2009)	61.5 (13.6)	—	—	Aerobic fitness (VO_2_max) (mL/kg/min) mean (SD): 14.7 (4.8); Fugl-Meyer sensorimotor test, mean (SD): 77.5 (25.0); Berg Balance Scale, mean (SD): 39.6 (12.3); Get Up and Go test, mean (SD): 21.3 (29.4)	MMSE, mean(SD):28.6(1.84)	—	—	—
Rosenfeldt et al. (2019)	56 (13.2)	Months post occurrence, mean (SD): 17.6 (4.3)	—	Fugl-Meyer Assessment, mean (SD): 34.6 (9.5); Stroke Impact Scale, physical composite, mean (SD): 57.2 (11.5)	Stroke Impact Scale, cognitive composite, mean (SD):83.6 (18.5)	Depression (measured by CES-D), mean(SD):12(9.2);	—	—
Tang et al. (2014)	66.4 (7.1)	Years post occurrence, mean (SD): 4.2 (2.9); Stroke type, lacunar/ ischemic/hemorrhagic/unknown, *n*:7/19/16/8; Stroke location, cortical/subcortical/brainstem/unknown, *n*:10/14/14/12; Limbs affected, R/L/Bilateral, *n*:18/31/1; National Institute of Health stroke scale, mean (SD): 1.5 (2.6)	BMI, mean (SD): 27.6(4.7); Resting systolic blood pressure (mmHg), mean (SD): 122 (12.3); Resting diastolic blood pressure (mmHg), mean (SD): 67.2 (6.9); Pulse pressure (mmHg), mean (SD): 54.5 (10.7)	Aerobic fitness (VO_2_max) (mL/kg/min) mean (SD): 16.9 (5.1); 6-min walk test, mean (SD): 300.2 (136.1). Berg Balance Scale score, mean (SD): 50.5 (3)	MoCA, mean(SD):25(3)	Center for Epidemiologic Studies – Depression scale, mean(SD):5.2(3.2)	Diabetes(all type2), n:38;	Smoking status, never/ former/ current, n:25/23/2;Total cholesterol(mmol/L), mean(SD):4.4(0.9); LDL cholesterol(mmol/L), mean(SD):2.4(0.7); HDL cholesterol(mmol/L), mean(SD):1.3(0.4); Triglycerides(mmol/L), mean(SD):1.5(0.6); Fasting glucose (mmol/L), mean(SD):5.4(1.3); Homocysteine(μmol/L), mean(SD):13.7(5.1)
Bo et al. (2019)	65.9 (2.7)	—	BMI, mean (SD): 27.9 (5.2); Resting heart rate, mean (SD): 70.6 (7.3)	—	MMSE, mean (SD): 16.7 (6)	—	—	education years, mean(SD): 4.9(1.4)

*ACER, Addenbrooke's Cognitive Examination-Revised; BMI, body mass index; CES-D: Center for Epidemiologic Studies depression scale; HADS-D, Hospital Anxiety and Depression Scale–Depression subscale; MMSE, Mini-Mental State Examination; MoCA, Montreal Cognitive Assessment; NIHSS, National Institutes of Health Stroke Scale; PSQI, Pittsburgh sleep quality index; VO_2_max, maximum oxygen intake*.

### Quality Assessment

**Figure 2:**
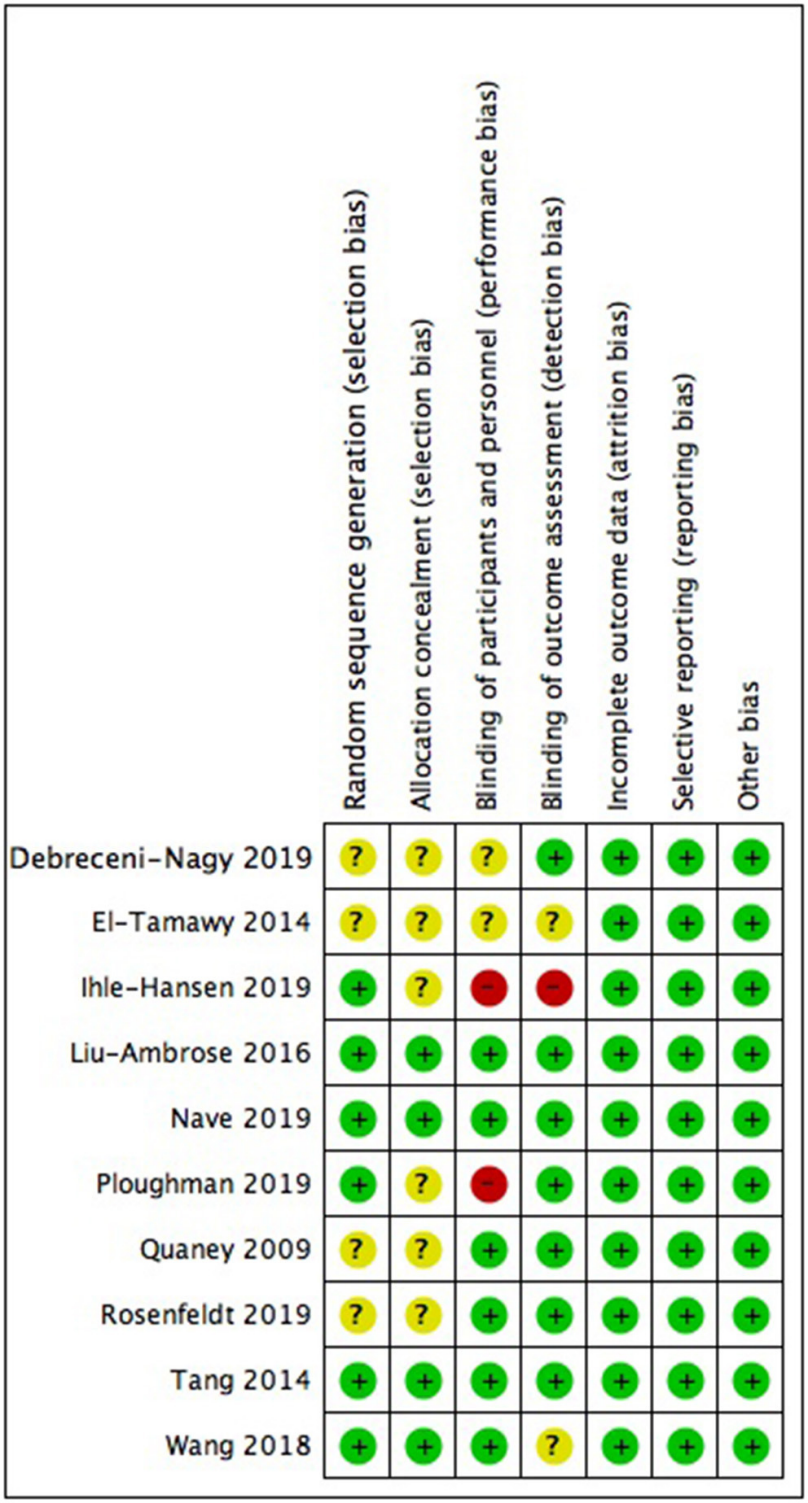
Risk of bias analysis in accordance with Cochrane Handbook.

The quality assessment is summarized in Figure 2. Nearly half of the studies did not report the method of sequence generation and allocation concealment and were judged to have an unclear risk of bias in these two regions. In all studies assessed, it was impossible to blind participates and therapists. Among them, six studies were judged that the outcome was unlikely to be interfered by absence of blinding. All studies gave information about drop-out and thus were considered having a low risk of bias in outcome reporting. Other domains were judged having a low risk of bias.

### Effects of Aerobic Exercise on General Cognitive Function

**Figure 3:**
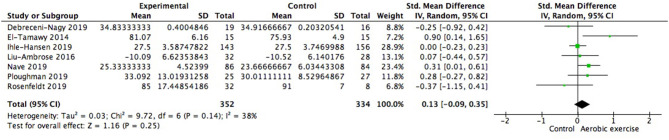
Meta-analysis of effect on global cognitive function of aerobic exercise.

**Figure 4:**
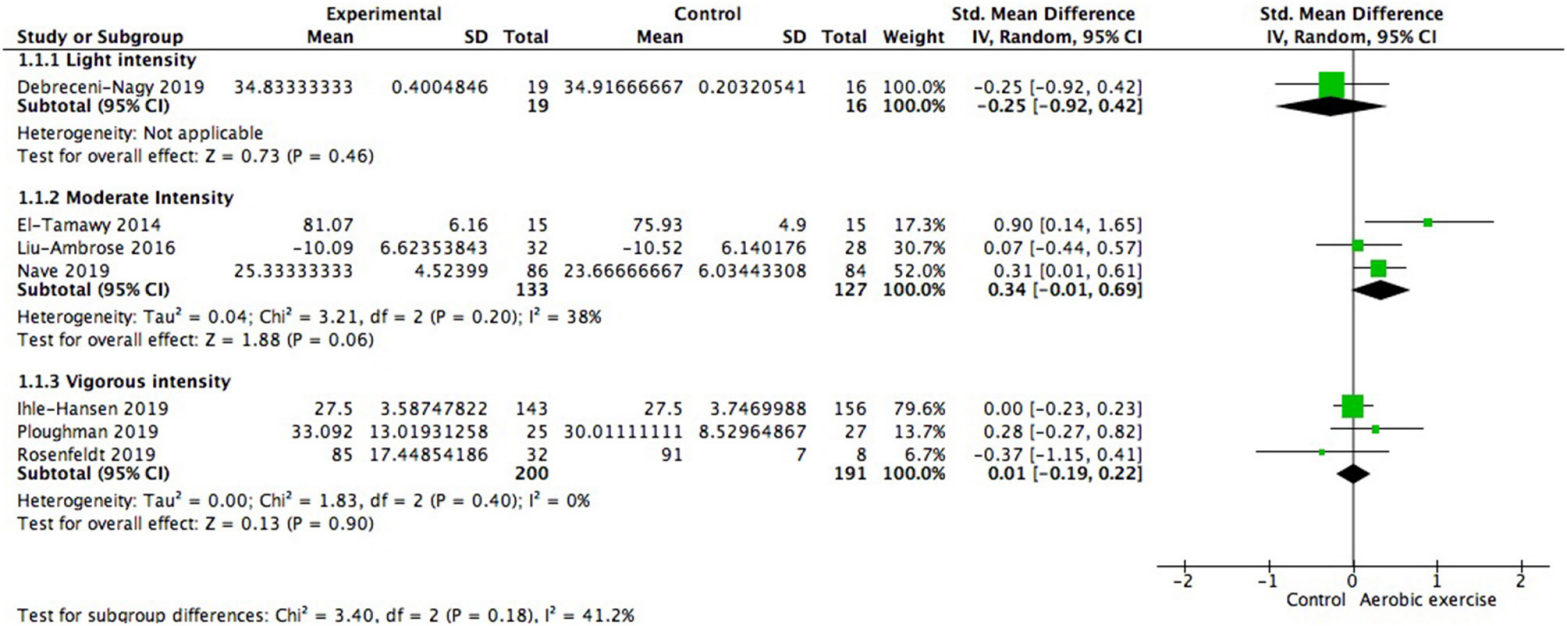
Subgroup analysis of whether exercise intensity could moderate global cognitive benefits.

Using data from seven studies, we first investigated whether patients with ischemic cerebrovascular disorder benefited from aerobic exercise in global cognitive function relative to controls. Results indicated a positive but not significant effect of aerobic exercise on general cognition (0.13; 95% Cl −0.09 to 0.35; *p* = 0.25; Figure 3). The assumption of homogeneity was valid among the studies [Chi^2^ = 9.72, df = 6(*p* = 0.14); *I*^2^ = 38%]. There was no significant change when any one of the study was removed. We then did the subgroup analysis based on exercise intensity. The findings revealed that training with moderate intensity could bring along cognitive benefits (0.34; 95% Cl −0.01 to 0.69; *p* = 0.06; Figure 4) while the performance in the vigorous-intensity group was less fascinating (0.01; 95% Cl −0.19 to 0.22; *p* = 0.9). Nevertheless, there was no significant subgroup differences [Chi^2^ = 3.4, df = 2(*p* = 0.18); *I*^2^ = 41.2%].

**Figure 5:**
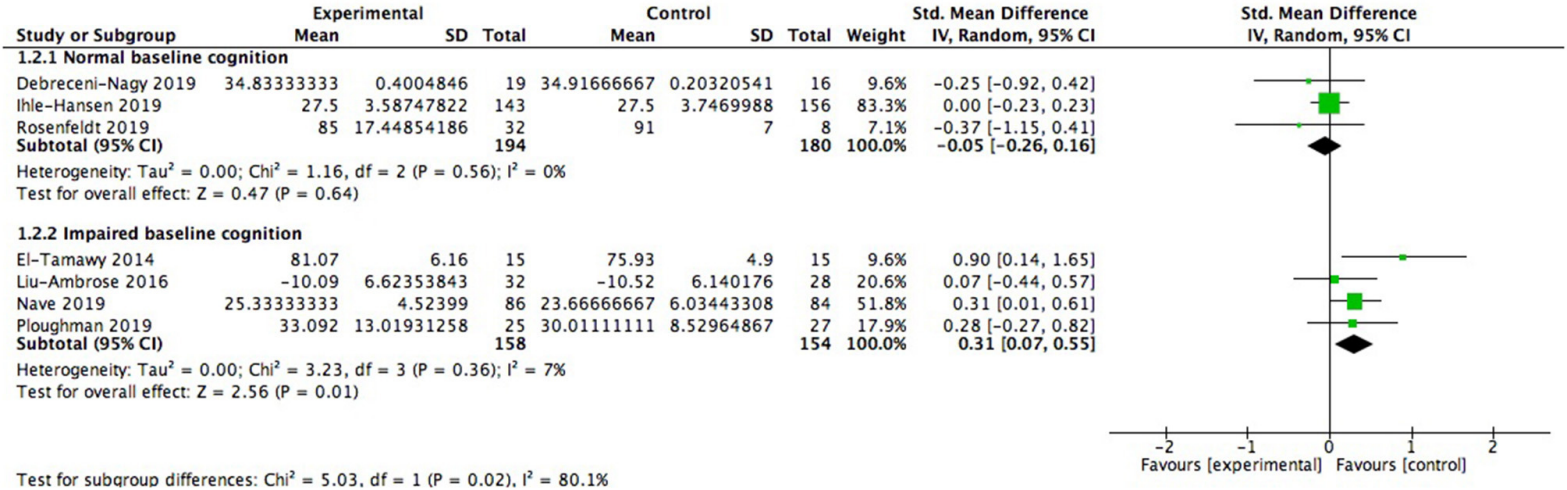
Subgroup analysis of modulation of baseline cognitive status on global cognitive function after aerobic exercise.

We also did subgroup analysis to explore whether baseline cognitive status exerted impacts on cognitive improvement. It was observed that patients already with cognitive impairment (312 participants) embraced significant cognitive benefits from aerobic exercise (0.31; 95% Cl 0.07–0.55; *p* = 0.01; Figure 5), while the effect size in patients with healthy baseline cognitive status (374 participants) failed to show this merit (−0.05; 95% Cl −0.26 to 0.16; *p* = 0.64). And there was significant difference between subgroups [Chi^2^ = 5.03, df = 1(*p* = 0.02); *I*^2^ = 80.1%].

**Figure 6:**
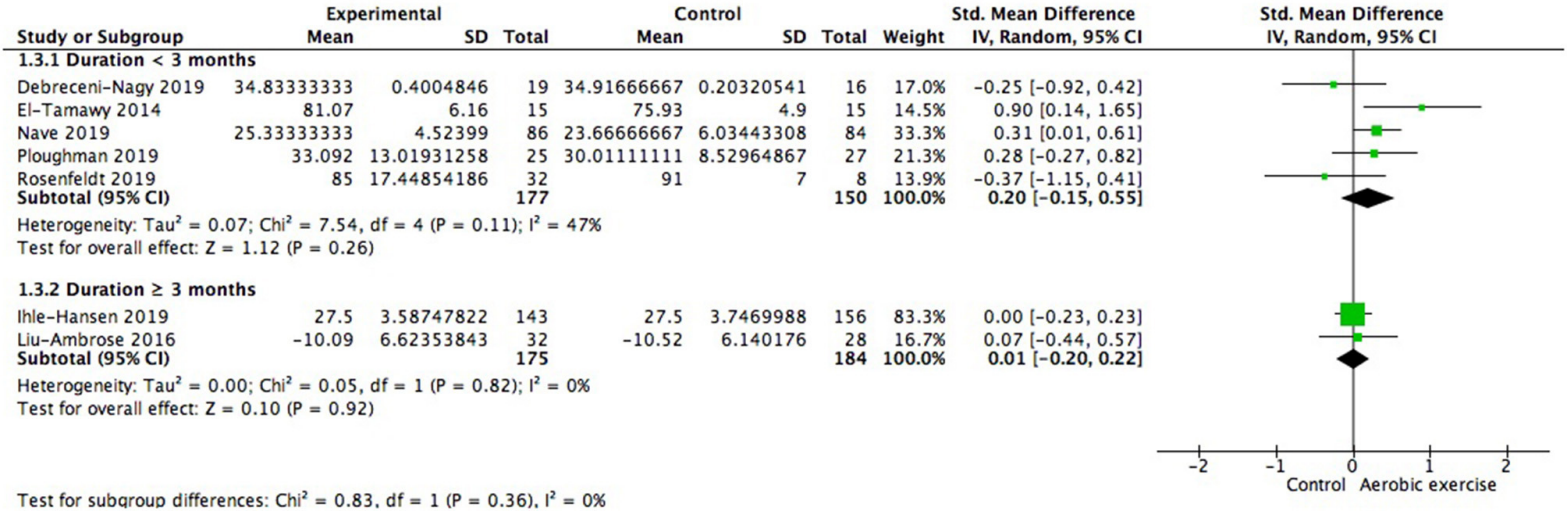
Subgroup analysis of moderating effect of exercise duration on global cognitive function.

In the studies lasting for more than 3 months (359 participants), one study went on for 6 months and the other lasted for 18 months. Among the studies lasting for < 3 months (327 participants), one lasted for 2.5 months, two lasted for 2 months, and the rest two lasted for 1 month. Both subgroups showed positive but not significant gains in global cognition (Figure 6). The effect size of shorter duration was larger. There was no significant difference between subgroups [Chi^2^ = 0.83, df = 1 (*p* = 0.36), *I*^2^ = 0%].

**Figure 7:**
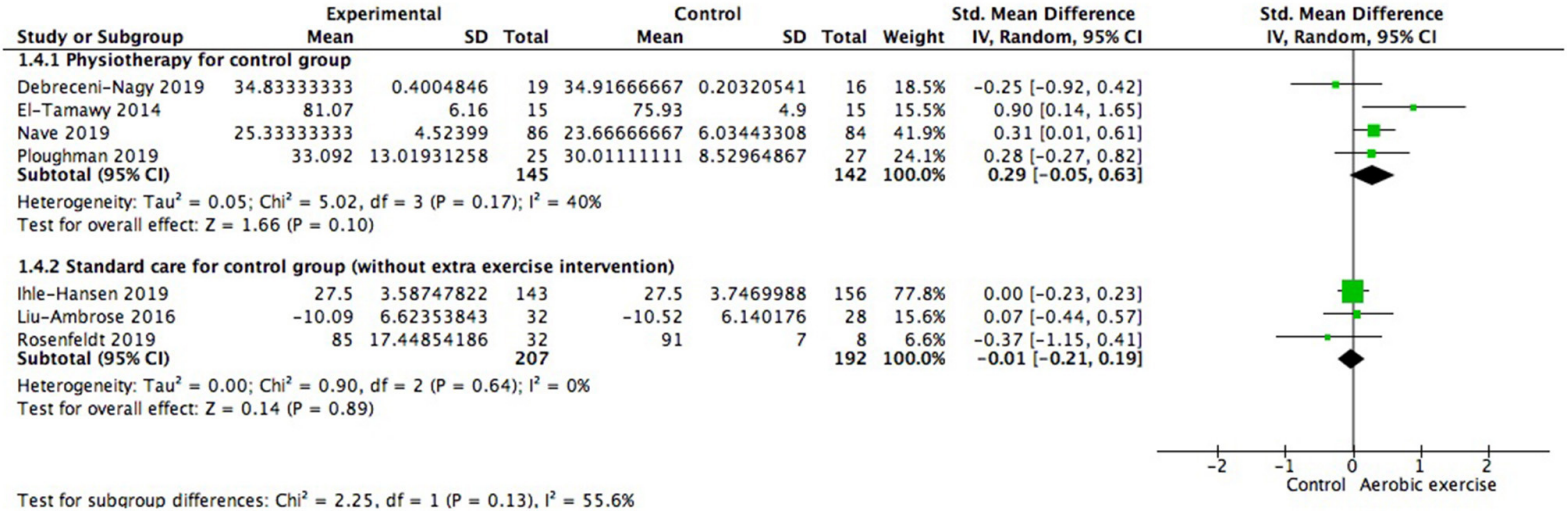
Subgroup analysis of whether strategies conducted in control group could impact the magnitude of effect sizes when measuring global cognitive gains.

Meanwhile, we also investigated the influence of different control conditions on cognition improvement after aerobic exercise. Four of the seven studies gave participants in control group physiotherapy (for example, stretching exercise) for alternative (287 participants) while the rest were only provided with usual care (e.g., education material without special focus on physical training, 399 participants). Results indicated that the adoption of physiotherapy or education program for control group was not the variant affecting the cognitive consequence [test for subgroup difference: Chi^2^ = 2.25, df = 1 (*p* = 0.13), *I*^2^ = 55.6%; Figure 7].

**Figure 8:**
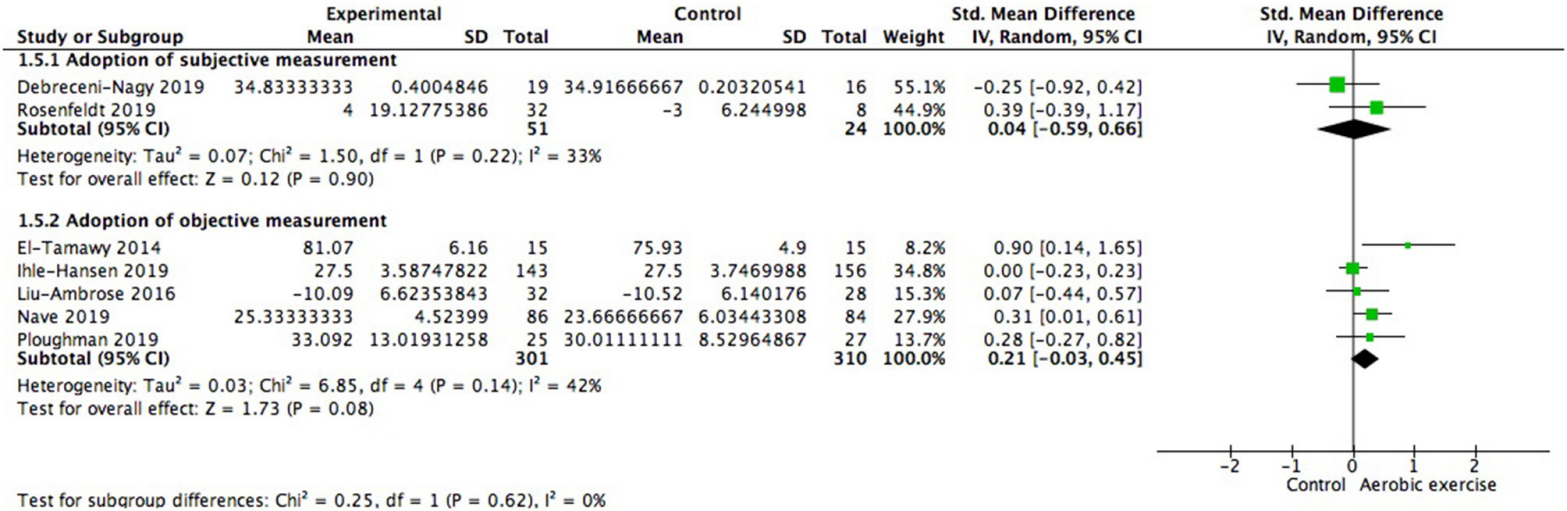
Subgroup analysis of whether type of measurement adopted was a moderating variable of global cognitive gains.

Moreover, we investigated whether subjective measurement would show variable consequence compared to objective measurement. We did not find significant difference between subgroups [Chi^2^ = 0.25, df = 1(*p* = 0.62); *I*^2^ = 0%; Figure 8]. Statistically homogenous was met in all subgroups.

**Figure 9:**
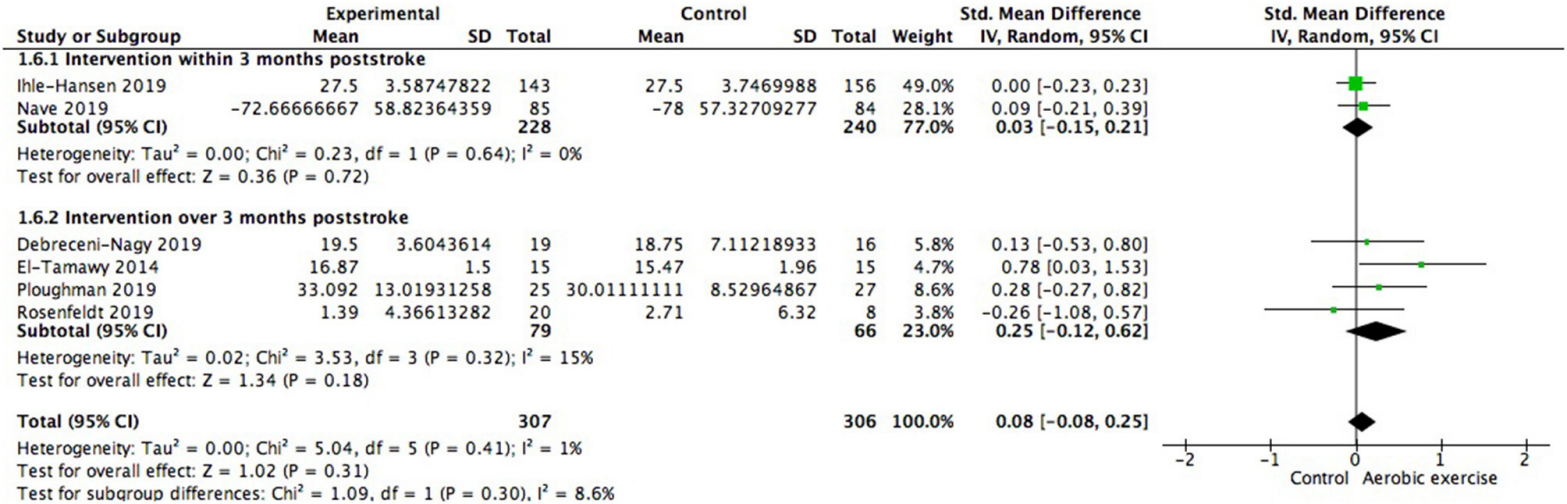
Meta-analysis of global cognitive growths from aerobic training in stroke survivors and the modulation of time interval between stroke occurrence and intervention on global cognitive benefits.

When the type of disease was restricted to stroke, cognitive gains were also positive but not significantly different from zero (0.08; 95% Cl −0.08 to 0.25; *p* = 0.31; Figure 9). We further investigated whether starting time of intervention poststroke exerted impacts on cognition. Among the 2 studies launched within 3 months poststroke (468 participants), one included patients who were 3 months post stroke and the other began 5–45 days (28.51 ± 17.24 days) after the occurrence. Results showed no significant between group difference [Chi^2^ = 1.09, df = 1 (*p* = 0.30); *I*^2^ = 8.6%].

### Effects of Aerobic Exercise on Processing Speed and Executive Function

**Figure 10:**
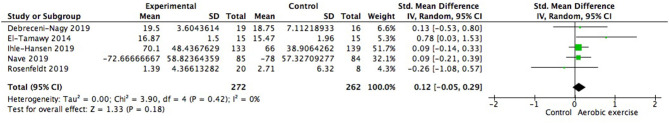
Meta-analysis of effect of aerobic training on cognitive processing speed.

**Figure 11:**
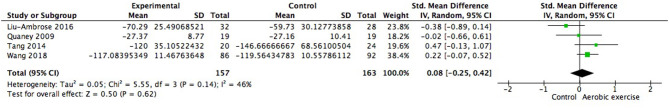
Meta-analysis of influence of aerobic training on response inhibition.

**Figure 12:**
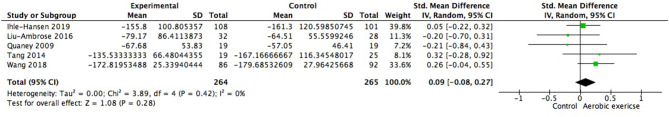
Meta-analysis of impact of aerobic exercise on set shifting.

**Figure 13:**
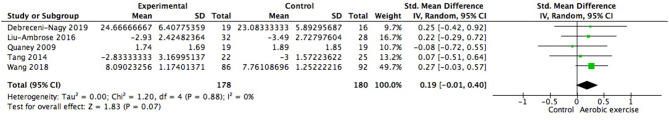
Meta-analysis of effect of aerobic exercise on working memory.

We also examined changes on special cognitive domains. The study of Debreceni-Nagy et al. reported results of two subtests of processing speed (coding subset and symbol search sum score) (Debreceni-Nagy et al., 2019). We applied the symbol search sum score in Figure 10 and the finding was similar if the coding subset was adopted. No significant between group difference was found in processing speed (0.12; 95% Cl −0.05 to 0.29; *p* = 0.18). Response inhibition, set shifting and working memory were three important aspects of executive function. However, none of these aspects showed significant difference from zero (Figures 11–13).

## Discussion

Our results suggested that aerobic exercise might improve cognitive function in patients with ischemic cerebrovascular disorder. Particularly, moderate intensity aerobic training might be the most promising mode to alleviate or prevent vascular cognitive impairment. Population already with cognitive impairment were suggested to benefit more from aerobic training compared to their cognitively healthy counterparts.

A previously published meta-analysis demonstrated a significant and moderate improvement in cognition after physical activity in stroke survivors (Oberlin et al., 2017). However, only 3 studies using aerobic exercise as the sole intervention were available in that study. A systematic review limited the intervention to aerobic exercise and suggested its positive effects on cognition post stroke (Zheng et al., 2016). However, the research team of this analysis also included studies where interventions like yoga, tai, and chi were employed. In our study, stricter criteria were employed to ensure that aerobic exercise was the sole intervention. We also investigated the influence of exercise intensity, duration, and starting point poststroke on cognitive gains, which might better guide future clinical practice. Specific domains including processing speed and executive function were studied, providing a deeper and broader sight into this issue.

Previous meta-analysis failed to find any significant improvement in global cognition after aerobic exercise in healthy old adults (Young et al., 2015; Sanders et al., 2019). However, population suffering from cognition impairment were suggested positively benefited from this intervention (Song et al., 2018; Sanders et al., 2019). Similarly, in our study, better improvement was observed in cognitively defective group. Aerobic exercise benefits cognition probably through the up-regulation of growth factors including BDNF, IGF-1, and VEGF, promoting neurogenesis and angiogenesis, especially in hippocampus (Cotman et al., 2007). Evidence suggested that the concentration of BDNF was positively correlated with Barthel index scores, a measure of ability for daily living. BDNF was also relevant to the concentration subscale of Mini-mental State Examination (MMSE) score despite no close association found between BDNF concentration and total MMSE score (Navarro-Martínez et al., 2015). IGF-1 was suggested as the possible biomarker of cognition and the decline of this molecule was related to cognitive impairment (Frater et al., 2018). In the model of vascular cognitive impairment, IGF-1 and IGF-1 mRNA were found downregulated in hippocampus (Gong et al., 2012). It has also been found in animal model that the inhibition of VEGF receptor would induce neurological injury, limiting the excretion of BDNF (Chen et al., 2019). It is possible that the reduction of these important growth factors in cognitively impaired participants makes them show greater responsiveness to aerobic exercise.

For stroke survivors, exercise with an intensity of 40–70% HRR or 50–80% maximum heart rate is recommended (Billinger et al., 2014). Our results showed that aerobic exercise with moderate intensity was the most promising mode to promote global cognitive gains in patients with ischemic cerebrovascular disorder. The study included using low intensity intervention (Debreceni-Nagy et al., 2019) did not show significant difference from zero. Several researches have revealed that cognitive benefit derived from physical exercise is does-dependent. The underlying mechanism is that elevated exercise intensity is positively correlated with increase of BDNF and reduction of pro-inflammatory cytokines (Manuela Crispim Nascimento et al., 2014; Cefis et al., 2019). And light aerobic exercise may not be sufficiently intense to induce the changes. It is also worth considering that Debreceni-Nagy employed severely deconditioned patients in the program and used subjective measurement in his research, possibly resulting in less cognitive gain. In the meantime, although the effect size of general cognitive function was negative, Debreceni-Nagy found that of working memory and processing speed was positive. Recent study also indicate low-intensity training is beneficial for executive function and cortical excitability (Morris et al., 2020), so it is still recommended for the deconditioned survivors. However, in our analysis, vigorous-intensity exercise did not return cognitive benefits, either. The diversity of participants' performance might account for the insignificance. Patients with ischemic cerebrovascular disease are usually characterized with reduced cardiovascular fitness (MacKay-Lyons and Makrides, 2002) and impaired motor or balance ability (Askim et al., 2014). Consequently, they may fail to reach the targeted intensity to optimize its efficacy. When considering duration, longer program duration did not predict more cognitive gains. This finding is similar to a previous meta-analysis (Northey et al., 2018). The possible reason is that the compliance might decline as the project goes on. Ihle-Hansen's research also found increased adherence to the intervention was significantly associated with up-regulated MMSE scores (Ihle-Hansen et al., 2019). Therefore, effort is needed to motivate the patients to engage in aerobic exercise in the long term. At present, no definite threshold has been found where the cognitive improvement of aerobic exercise could occur. Intensity, total duration, time for every session and frequency are all factors needed further exploration to reach the counterbalance for the optimal dose of exercise. In stroke survivors, the time point to initiate the exercise program was not the predictor of cognitive gains although the effect size of later intervention was greater. Pruski from Johns Hopkins proposed that the first 3 months post stroke were the most important period for recovery where most improvement could occur. And after 6 months, the progress would be much slower and reach a steady stage (Johns Hopkins Medicine, 2020). However, the stable condition and recovery of motor function may account for the benefits of late intervention. Moreover, the objective measurement might be the better choice when evaluating the cognitive gains, which is in line with the previous meta-analysis (Oberlin et al., 2017).

Processing speed refers to the time interval between sensory stimulus and related behavior response (Jensen, 2006). Previous trials indicated better aerobic endurance was correlated to improved processing speed and executive function in healthy old adults (Zettel-Watson et al., 2015). Aerobic exercise was also found beneficial for processing speed in patients with mild cognitive impairment (Zhu et al., 2018). In a study investigating multiple sclerosis, processing speed was positively associated with aerobic capacity which was indicated by VO_2max_ and balance (Sandroff and Motl, 2012). Also in multiple sclerosis, another study indicated that increased regional gray matter volumes in medial frontal gyrus, anterior cingulate cortex, and the precuneus were associated with the improved processing speed after aerobic exercise (Prakash et al., 2010). Executive function is a set of cognitive processes essential for behavior control to reach certain goals, for example, planning, organizing, coordinating and problem-solving (Diamond, 2013). Executive function relies on the integrity of prefrontal and parietal circuits (Colcombe and Kramer, 2003). Later report also demonstrated reduced gray volume in these regions in aged population and aerobic exercise could eliminate this aged-related deterioration (Colcombe et al., 2003). Another study demonstrated similar result as the cortical thickness increased in left caudal middle frontal area (Stern et al., 2017). Increased neural activity was also found in middle frontal gyrus, superior frontal gyrus, and the superior and inferior parietal lobules in aerobically trained older adults, together with reduced activity in the dorsal region of anterior cingulate cortex which was related to behavior conflicts (Colcombe et al., 2004). Nevertheless, in our study, none of the effect size in processing speed and executive function showed significant difference from zero. Liu-Ambrose's group also conducted the test of EXIT-25, an assessment of global executive function and did not find any improvement after aerobic training (Liu-Ambrose et al., 2016). Considering the special role of relevant lobes in processing speed and executive function, we proposed that the location of vascular disorder might be one variant involved. However, none of the included studies reported such information and thus it is needed settling in future investigation. Another factor which might get involved in cognitive benefits of these specific aspects induced by aerobic exercise was sex. A meta-analysis demonstrated greater cognitive gains in studies with over 50% participant population being women, indicating that women could benefit more from aerobic exercise in cognition compared to their male counterparts (Colcombe and Kramer, 2003). Similarly, another meta-analysis has found better executive function performance in studies with higher percentage of women after aerobic training (Barha et al., 2017). Baker's group investigated sex-specific effect of aerobic exercise in old adults with mild cognitive impairment. Men and women showed similar improvement in Trail B Test and Task Switching, the tests designed for set shifting performance. For Stroop Test (test for response inhibition) and Symbol Digit Modalities Test (test for processing speed), however, only women gained benefits while aerobic training had no effect on men (Baker et al., 2010). In our study, all trials included for analysis of processing speed and executive function are male dominant except the one conducted by Liu-Ambrose et al. (2016). And there were more men when considering the total number of participants. The secondary analysis of Tang's study also found female stroke survivors benefited more in set shifting and response inhibition (Khattab et al., 2019). Females had greater progression in white matter hyperintensity while this change in males was not significant according to report from Liu-Ambrose (Dao et al., 2019). Among the underlying factors, the difference in BDNF level might be of important influence. Animal models suggested positive effect of estrogen on BDNF (Blanc et al., 2010). The gene coding BDNF contains a sequence similar to the target of estrogen. The reduction of BDNF mRNA could be rescued by estrogen supplement in ovariectomized animals (Singh et al., 1995; Sohrabji et al., 1995). In the case of global cerebral ischemia, the extranuclear estrogen receptors get involved in ERK-Akt-CREB-BDNF signaling and activation of this pathway is associated with neuroprotection and cognitive preservation (Yang et al., 2010). Women have higher plasma BDNF level. Lower level of BDNF is related to further reduction of whole brain and frontal white matter volumes only in women (Komulainen et al., 2008; Driscoll et al., 2012). The secondary analysis of study of Liu-Ambrose et al. also showed increased level of BDNF level in women but decreased concentration in men (Barha Cindy et al., 2017).

However, these results should be interpreted carefully, as some effect size was evaluated from small number of studies, limiting the precision of pooled effects. Meanwhile, we restricted the study language to English, which might cause bias in this review. In addition, in this analysis, stroke is still the main type of disease studied and more trials should be conducted in other types of ischemic cerebrovascular disease to establish a comprehensive understanding of the cognitive benefits of aerobic exercise in such kind of disorder. Moreover, other factors like type, location and severity of stroke, education status, motor deflects were only available in a few studies, making it difficult for us to investigate whether they might impact on effect size. Only four of the studies included reported attendance and compliance of intervention. And we considered it great value to study on the influence of participants' adherence to training program. Ultimately, it is worthy of effort to explore gains in other cognitive domains (for example, verbal fluency and language) after aerobic training. Future studies are needed to confirm and expand our findings.

## Conclusion

The results of our analysis indicated that aerobic exercise might exert positive effects on cognition in ischemic cerebrovascular disorder survivors, especially for those already with cognitive impairment. Moderate intensity training tends to be the applicable selection. Duration of the training program and the initiation time of exercise of stroke survivors were not the predictors for cognitive gains. Our study did not find favorable effects of aerobic exercise on executive function and processing speed. Future work is needed to explore the optimal exercise parameters and benefiting population.

## Data Availability Statement

The datasets presented in this study can be found in online repositories. The names of the repository/repositories and accession number(s) can be found in the article/supplementary material.

## Author Contributions

YS contributed to conception and design of the study. YS, QH, and YX conducted the database search, study selection, data collection, and extraction. Data analysis was performed by QH and WZ. YS, QH, and SZ wrote the manuscript. All authors contributed to manuscript revision, read, and approved the submitted version.

## Conflict of Interest

The authors declare that the research was conducted in the absence of any commercial or financial relationships that could be construed as a potential conflict of interest.
